# Collective Response to Media Coverage of the COVID-19 Pandemic on Reddit and Wikipedia: Mixed-Methods Analysis

**DOI:** 10.2196/21597

**Published:** 2020-10-12

**Authors:** Nicolò Gozzi, Michele Tizzani, Michele Starnini, Fabio Ciulla, Daniela Paolotti, André Panisson, Nicola Perra

**Affiliations:** 1 University of Greenwich London United Kingdom; 2 ISI Foundation Torino Italy; 3 Quid Inc San Francisco, CA United States

**Keywords:** social media, news coverage, digital epidemiology, infodemiology, infoveillance, infodemic, digital epidemiology, data science, topic modeling, pandemic, COVID-19, Reddit, Wikipedia, information, response, risk perception, behavior

## Abstract

**Background:**

The exposure and consumption of information during epidemic outbreaks may alter people’s risk perception and trigger behavioral changes, which can ultimately affect the evolution of the disease. It is thus of utmost importance to map the dissemination of information by mainstream media outlets and the public response to this information. However, our understanding of this exposure-response dynamic during the COVID-19 pandemic is still limited.

**Objective:**

The goal of this study is to characterize the media coverage and collective internet response to the COVID-19 pandemic in four countries: Italy, the United Kingdom, the United States, and Canada.

**Methods:**

We collected a heterogeneous data set including 227,768 web-based news articles and 13,448 YouTube videos published by mainstream media outlets, 107,898 user posts and 3,829,309 comments on the social media platform Reddit, and 278,456,892 views of COVID-19–related Wikipedia pages. To analyze the relationship between media coverage, epidemic progression, and users’ collective web-based response, we considered a linear regression model that predicts the public response for each country given the amount of news exposure. We also applied topic modelling to the data set using nonnegative matrix factorization.

**Results:**

Our results show that public attention, quantified as user activity on Reddit and active searches on Wikipedia pages, is mainly driven by media coverage; meanwhile, this activity declines rapidly while news exposure and COVID-19 incidence remain high. Furthermore, using an unsupervised, dynamic topic modeling approach, we show that while the levels of attention dedicated to different topics by media outlets and internet users are in good accordance, interesting deviations emerge in their temporal patterns.

**Conclusions:**

Overall, our findings offer an additional key to interpret public perception and response to the current global health emergency and raise questions about the effects of attention saturation on people’s collective awareness and risk perception and thus on their tendencies toward behavioral change.

## Introduction

### Background

In the next influenza pandemic, be it now or in the future, be the virus mild or virulent, the single most important weapon against the disease will be a vaccine. The second most important will be communication.

This evocative sentence was written in May 2009 by John M Barry [1] in the early phases of what would soon become the 2009 H1N1 pandemic. In his essay, Barry summarized the mishandling of the deadly 1918 Spanish influenza, highlighting the importance of precise, effective, and honest information at the onset of health crises.

Eleven years later, the world is facing another pandemic. The cause is not a novel strain of influenza; however, unfortunately, Barry’s words are still extremely relevant. In fact, as SARS-CoV-2 spreads worldwide and a vaccine may still be far in the future, the most important weapons to reduce the burden of the disease are nonpharmaceutical interventions [2,3]. Social distancing has become paramount, gatherings have been cancelled, and mobility within and across countries has been dramatically reduced. While these measures have been enforced to different extents across nations, they all rely on compliance. The effectiveness of these measures is linked to risk and susceptibility perception [4]; thus, the information to which citizens are exposed is of fundamental importance.

History repeats itself, and humanity appears to not be able to learn from its past mistakes. As happened in 1918, despite early evidence from China [5,6], the virus was first equated by many people with common seasonal influenza. As in 1918, many national and regional governments organized campaigns aimed at encouraging social activities (and thus local economies) while actively attempting to convince people that their cities were safe and that the spread of the disease was isolated in faraway locations. For example, the hashtag #*MilanoNonSiFerma* (“Milan does not stop”) was coined to invite citizens in Milan to go out and live normally, while free aperitifs were offered in Venice. In hindsight, of course, it is easy to criticize the initial response in Italy. In fact, the country was one of the first to experience rapid growth of hospitalization [7]. However, the Mayor of London, 12 days before the national lockdown and a few days after the extension of the *cordon sanitaire* to the entire country in Italy, affirmed via his official Facebook page [8] that “we should carry on doing what we’ve been doing.” More generally, in several western countries, news reports from other countries reporting concerning epidemic outbreaks were not considered to be relevant to the local situation. This initial phase aimed at conveying low local risk and boosting confidence in national safety was repeated, at different times, across countries. A series of surveys conducted in late February provide a glimpse of the possible effects of these approaches. These surveys report that citizens of several European countries, despite the grim news coming from Asia, were overly optimistic about the health emergency, placing their risk of infection at 1% or less [9]. As in 1918, countries that reacted earlier rather than later were better able to control the virus, with significantly fewer infections [10-14].

History repeats itself; however, the context is often radically different. In 1918, news circulated slowly via newspapers, controlled by editorial choices; of course, news also spread by word of mouth. In 2009, we witnessed the first pandemic in the social media era. Newspapers and television were still very important sources of information; however, Twitter, Facebook, YouTube, and Wikipedia started to become relevant for decentralized news consumption, boosting of peer discussions, and spreading of misinformation. Currently, these platforms and websites are far more popular and integral parts of society, and they are instrumental sources of national and international news circulation. Together with traditional news media, these platforms and websites are the principal sources of information for the public. As such, they are fundamental drivers of people’s perception and opinions and thus of their behaviors. This is particularly relevant for health issues. For example, approximately 60% of adults in the United States consulted web-based sources to gather health information [15].

Furthermore, some platforms are acknowledging their growing responsibility in media consumption and have introduced specific features to increase users’ awareness and levels of information.

### Prior Work

With respect to past epidemics and pandemics, studies on traditional news coverage of the 2009 H1N1 pandemic highlighted the importance of framing and its effect on people’s perception, behaviors (eg, vaccination intent), and stigmatization of cultures at the epicenter of the outbreak, as well as how these factors differ across countries and cultures [16-21]. During the Zika virus epidemic in 2016, public attention was synchronized across US states, driven by news coverage about the outbreak and independent of the real local risk of infection [22]. With respect to the COVID-19 pandemic itself, a recent study clearly showed how Google searches for *coronavirus* in the United States spiked significantly immediately after the announcement of the first confirmed case in each state [23]. Several studies based on Twitter data have also highlighted how misinformation and low-quality information about COVID-19, although limited overall, spread before the local outbreak and rapidly expanded once local epidemics started [24-26]. In the current landscape, this spread of misinformation has the potential to encourage irrational, unscientific, and dangerous behaviors. On the other hand, despite some important limitations [27], modern media has become a key data source to observe and monitor health. In fact, posts on Twitter [28-33], Facebook [34], and Reddit [35,36], page views in Wikipedia [37,38], and searches on Google [39,40] have been used to study, nowcast, and predict the spreading of infectious diseases as well as the prevalence of noncommunicable illnesses. Therefore, in the current full-fledged digital society, information is not only key to inform people’s behavior but can also be used to develop an unprecedented understanding of these behaviors as well as of the phenomena driving them.

### Goal of This Study

The context in which COVID-19 is unfolding is very heterogeneous and complex. Traditional and social media are integral parts of public perception and opinions, and they have potential to trigger behavior changes and thus influence the spread of the pandemic. This complex landscape must be characterized to understand the public attention and response to media coverage. Here, we addressed this challenge by assembling a heterogeneous data set that includes 227,768 news reports and 13,448 YouTube videos published by traditional media, 278,456,892 views of topical Wikipedia pages, and 107,898 submissions and 3,829,309 comments from 417,541 distinct users on Reddit, as well as epidemic data in four different countries: Italy, the United Kingdom, the United States, and Canada. First, we explored how media coverage and epidemic progression influence public attention and response. To achieve this, we analyzed news volume and COVID-19 incidence with respect to volumes of Wikipedia page views and Reddit comments. Our results show that public attention and response are mostly driven by media coverage rather than by disease spread. Furthermore, we observed the typical saturation and memory effects of public collective attention. Moreover, using an unsupervised topic modeling approach, we explored the different topics framed in traditional media and in Reddit discussions. We show that while the attention of news outlets and internet users toward different topics is in good accordance, interesting deviations emerge in their temporal patterns. Also, we highlight that at the end of our observation period, general interest grew toward topics about the resumption of activities after lockdown, the search for a vaccine against SARS-CoV-2, acquired immunity, and antibody tests. Overall, the research presented here offers insights to interpret public perception and response to the current global health emergency and raises questions about the effects of attention saturation on collective awareness and risk perception and thus on tendencies toward behavioral change.

## Methods

### Data Set

#### News Articles and Videos

We collected news articles using News API, a service that allows free downloads of articles published on the internet in a variety of countries and languages [41]. For each country considered, we downloaded all relevant articles published on the internet by selected sources in the period from February 7 to May 15, 2020. We selected relevant articles by considering those citing one of the following keywords: *coronavirus*, *covid19*, *covid-19*, *ncov-19*, and *sars-cov-2*. Note that for each article, we could access the title, a description, and a preview of the full text. In total, our data set consisted of 227,768 news articles; 71,461 were published by Italian media, 63,799 by UK media, 82,630 by US media, and 9878 by Canadian media.

Additionally, we collected all videos published on YouTube by major news organizations in the four countries under investigation via their official YouTube channels using the official application programming interface (API) [42]. In this process, we downloaded the titles and descriptions of all the videos and selected as relevant those that mentioned one of the following keywords: *coronavirus*, *virus*, *covid*, *covid19*, *sars*, *sars-cov-2*, and *sarscov2*. The reach of each channel (measured by the number of subscribers) varied drastically, from more than 9 million for CNN (United States) to approximately 12,000 for Ansa (Italy). In total, the YouTube data set consisted of 13,448 videos; 3325 were published by Italian channels, 3525 by UK channels, 6288 by US channels, and 310 by Canadian channels.

It is important to underline that while there is good overlap between the sources of news articles and videos, some do not match. This is due to the fact that not all news organizations have a YouTube channel, while others do not produce traditional articles. In Multimedia Appendix 1, we provide a complete list of the news outlets and YouTube channels we considered.

#### Reddit Posts

Reddit is a social content aggregation website on which users can post, comment, and vote on content. It is structured in subcommunities (ie, subreddits) that are centered around a variety of topics. Reddit has already been proven to be suitable for a variety of research purposes, ranging from the study of user engagement and interactions between highly related communities [43,44] to postelection political analyses [45]. Moreover, it has been used to study the impact of linguistic differences in news titles [46] and to explore recent web-related issues such as hate speech [47] and cyberbullying [48] as well as health-related issues such as mental illness [49]; it also provides insights into the opioid epidemic [50].

We used the Reddit API to collect all submissions and comments published in Reddit under the subreddit r/Coronavirus from February 15 to May 15, 2020. After cleaning the data by removing entries deleted by authors and moderators, we retained only submissions with scores >1 to avoid spam. We removed comments with <10 characters and with >3 duplicates to avoid including automatic messages from moderators. The final data set contained 107,898 submissions and 3,829,309 comments from 417,541 distinct users.

To characterize the topics discussed on Reddit, we then selected entries with links to English-language news outlets. The contents of the URLs were extracted using the available implementation of the method described in [51], resulting in 66,575 valid documents.

Reddit does not provide explicit information about users’ locations; therefore, we used self-reporting via regular expression to assign locations to users. Reddit users often declare geographical information about themselves in submissions or comment texts. We used the same approach described in [50], in which the use of regular expressions was found to be reliable, resulting in high correlation with census data in the United States; however, we acknowledge a potential higher bias at the country level due to heterogeneities in Reddit population coverage and user demographics. We selected all English-language texts containing expressions such as “I am from” or “I live in” and extracted candidate expressions from the text that followed the expressions to identify texts that represented country locations. By removing inconsistent self-reporting, we were able to assign a country to 789,909 distinct users, among which 41,465 had posted at least one comment in the subreddit r/Coronavirus (13,811 from the United States, 6870 from Canada, 3932 from the United Kingdom, and 445 from Italy).

#### Wikipedia Page Views

Wikipedia has become a popular digital data source to study health information–seeking behavior [52] and to monitor and forecast the spreading of infectious diseases [53,54]. Here, we used the Wikimedia API [55] to collect the number of visits per day to Wikipedia articles and the total monthly visits to a specific project from each country. We considered language to be indicative of a specific country, suggesting that the relevant projects for our analysis would be written in English and Italian (ie, *en.wikipedia* and *it.wikipedia*, respectively). We chose articles directly related to COVID-19 and those in the “See also” section of each page at the time of the analysis (February 7 to May 15, 2020), including country-specific articles (see Multimedia Appendix 1 for the full list of webpages considered).

Except for Italian, where the language is highly indicative of the location, the number of visits to English pages is almost evenly distributed among English-speaking countries. To normalize the signal related to each country, we weighted the number of daily visits to a single article from a specific project *p*, *S_p_*(*d*), with the total number of monthly visits from a country *c*, to the related Wikipedia project 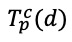, such that the number of daily page views for a given Wikipedia project and country is:


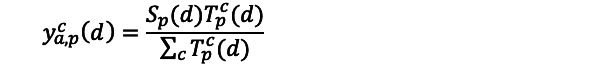
(**1**)

where the denominator is the total number of views of the specific Wikipedia project. The total volume of views on day *d* from country *c* is then given by the sum over all the articles *a* and projects *p*, namely:


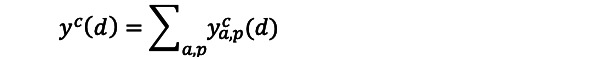
(**2**)

### Media Coverage and Collective Web-Based Response

With our data set, we aimed to provide an overview of media coverage and a proxy of public attention and response. On the one hand, the study of news articles and videos enabled us to estimate the exposure of the public to information about the COVID-19 pandemic in traditional news media. On the other hand, the study of users’ discussions and responses on social media (through Reddit) and information-seeking (through Wikipedia page views) allowed us to quantify the reaction of individuals both to the COVID-19 pandemic and to news exposure. As mentioned in the Introduction, previous studies showed the usefulness of social media, internet use, and search trends to analyze health-related information streams and monitor public reaction to infectious diseases [56-60]. Hence, we considered the volume of comments of geolocalized users on the subreddit r/Coronavirus to explore the public discussion in reaction to media coverage of the epidemic in the various countries; meanwhile, we considered the number of views of relevant Wikipedia pages about the COVID-19 pandemic to quantify users’ interest. It is important to stress that Reddit and Wikipedia provide different aspects of internet users’ behavior and collective response. In fact, while Reddit posts can be regarded as a general indicator of the web-based discussion surrounding the global health emergency, the number of visits to COVID-19–related Wikipedia pages is a proxy of health information–seeking behavior. Health information–seeking behavior is the act by which individuals retrieve and acquire new knowledge about a specific topic related to health [61,62]; it is likely to be triggered on a population scale by a disrupting event, such as the threat of a previously unknown disease [63,64].

### Linear Regression Approach to Model Collective Attention

To analyze the relationship between media coverage, epidemic progression, and users’ collective web-based response, we considered a linear regression model that predicts the public response for each country given the amount of news exposure. To include “memory effects” in the public response to media coverage, we also considered a modified version of this simple model, in which we weight a cumulative news articles volume time series with an exponential decay term [22]. Formally, we define the new variable as:


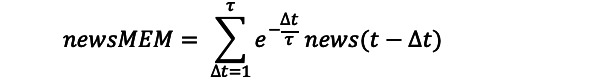
(**3**)

where *τ* is a free parameter that sets the memory time scale and is tuned by comparing different variants of the linear regression with *τ* ∈ [1,45] in terms of the adjusted coefficient of determination *R*^2^ [65] (results for the best *τ* are displayed). These two models were compared to a linear regression that considers only COVID-19 incidence to predict public collective attention. Then, the models considered are:

Model I: y_t_ = α_1_incidence_t_ + u_t_

Model II: y_t_ = α_1_news_t_ + u_t_

Model III: y_t_ = α_1_news_t_ + α_2_newsMEM_t_ + u_t_ (4)

where *y_t_* can be the volume of Reddit comments of geolocalized users or of country-specific Wikipedia visits, and *u_t_* is the error term. In Multimedia Appendix 1, we provide more details on the model diagnostics and fitting procedure.

### Topic Modeling

Topic modeling has emerged as one of the most effective methods for classifying, clustering, and retrieving textual data, and it has been the object of extensive investigation in the literature. Many topic analysis frameworks are extensions of well-known algorithms that are considered to be state-of-the-art for topic modeling. Latent Dirichlet allocation (LDA) [66] is the reference for probabilistic topic modeling. Nonnegative matrix factorization (NMF) [67] is the counterpart of LDA for matrix factorization. Although there are many approaches to temporal and hierarchical topic modeling [68-70], we chose to apply NMF to the data set and then build time-varying intensities for each topic using the publication dates of the articles. Starting from a data set *D* containing the news articles shared in Reddit, we extracted words and phrases with the methodology described in [71], discarding terms with frequencies >10, to form a vocabulary *V* with approximately 60,000 terms. Each document was then represented as a vector of term counts in a bag-of-words approach. We applied term frequency–inverse document frequency (TF-IDF) normalization [72] and extracted a total of *K*=64 topics through NMF:


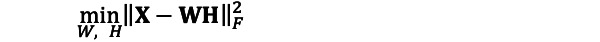
 (**5**)

where 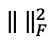 is the Frobenius norm and **X** ∈ **R**^|^*^D^*^| × |^*^V^*^|^ is the matrix resulting from TF-IDF normalization, subject to the constraint that the values in **W** ∈ **R**^|^*^D^*^| ×^ *^K^* and **H** ∈ **R***^K^* ^× |^*^V^*^|^ must be nonnegative. The nonnegative factorization was achieved using the projected gradient method with sparseness constraints, as described in [73,74]. The matrix H was then used as a transformation basis for other data sets (eg, with a new matrix 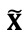, we fixed H and calculated a new 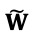 according to Equation 5). For each topic *k*, we built a time series *s_k_* for each data set *D*, where 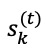 is the strength of topic *k* at time *t*. For the news outlets data set, 
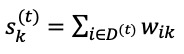
, where *D*^(^*^t^*^)^ is the set of all documents shared at time *t* in news outlets. For Reddit, we weighted each shared document by its number of comments, and 
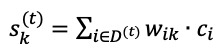
, where *D*^(^*^t^*^)^ is the set of all documents shared at time *t* in Reddit and *c_i_* is the number of comments associated with document *i*. Finally, we defined the relevance *R* of a topic as the integral in time of the strength. Therefore, given *t*_0_ and *t_f_* as the start and end of our analysis interval, 
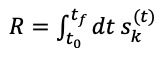
. In Multimedia Appendix 1, we show that choosing *K*=64 as the number of extracted topics provides a good balance between sufficient captured topic strength and good topic coherence.

## Results

### Impact of Media Coverage and Epidemic Progression on Collective Attention

**Figure 1:**
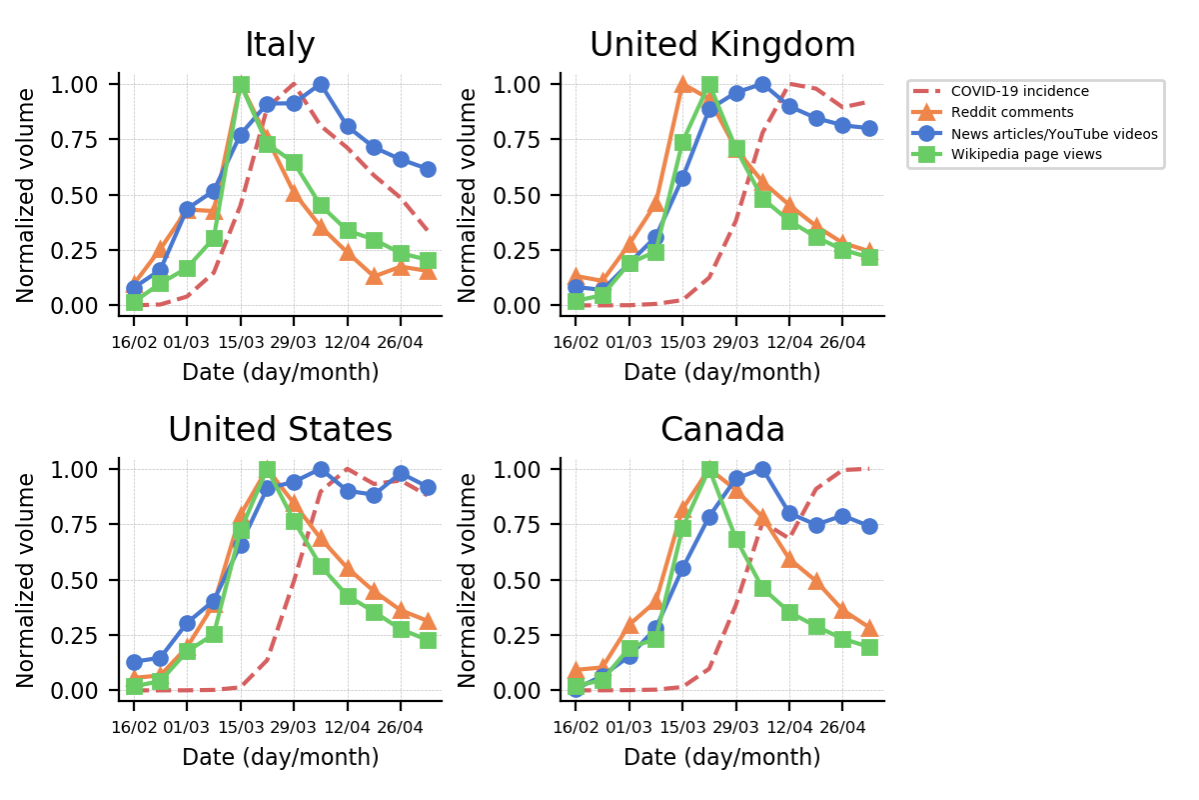
Normalized weekly volumes of news articles and YouTube videos, Reddit comments, and Wikipedia page views related to the COVID-19 pandemic and the incidence of COVID-19 in different countries.

To answer the important question of how collective attention is shaped by news media coverage and epidemic progression, we started by comparing the weekly volumes of news stories and videos published on YouTube, Wikipedia page views, and Reddit comments of geolocalized users with the weekly COVID-19 incidence in the four countries considered (Figure 1). It can be seen that as COVID-19 spread, both media coverage and public interest grew with time. However, public attention, quantified by the number of Reddit comments and Wikipedia page views, sharply decreased after reaching a peak, even though the volume of news stories and the incidence of COVID-19 remained high. Furthermore, the peak in public attention consistently anticipated the maximum media exposure and maximum COVID-19 incidence.

The correlation between media coverage, public attention, and progression of the epidemic is quantified in more detail in Table 1. The table shows that news coverage of each country is strongly correlated with COVID-19 incidence (both global and domestic) and slightly less correlated with the volumes of Reddit comments and Wikipedia views, which in turn are much less correlated with COVID-19 incidence (both global and domestic). This result was observed for all countries under consideration; it highlights how the spread of COVID-19 triggered media coverage as well as how public response was more likely to be driven by news exposure in each country than by the progression of COVID-19.

**Figure 2:**
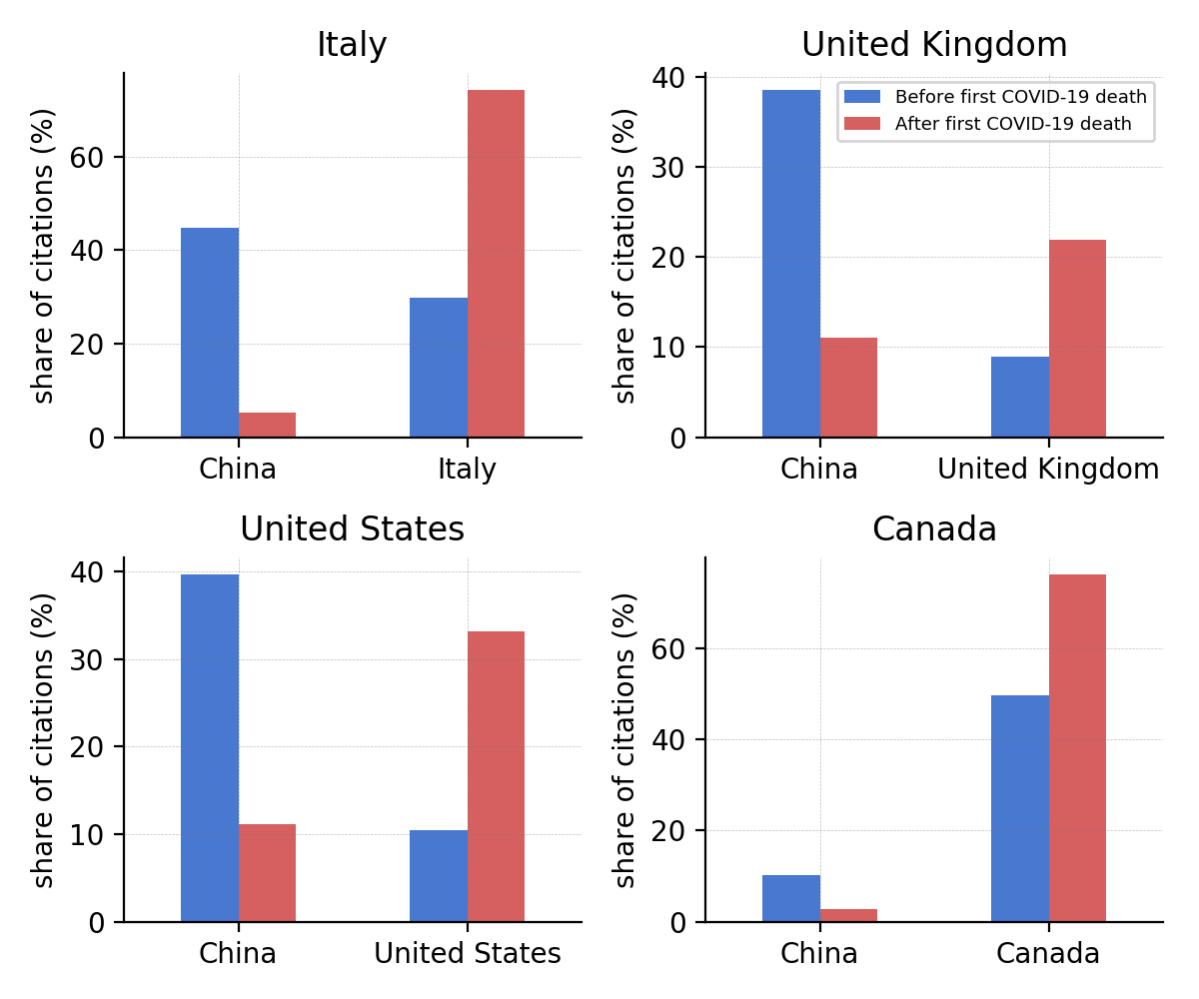
Shares of citations of China versus home country locations by Italian, UK, US, and Canadian news outlets before and after the first COVID-19 death occurred in each country.

Beyond these observations, Figure 2 shows the share of citations of Chinese versus home country locations by Italian, UK, US, and Canadian news outlets before and after the first COVID-19 death occurred in those countries; the geographic locations were extracted from the text using the methods described in [75,76]. Interestingly, Italy is the only country where the news volume shows a higher correlation with domestic incidence than with global incidence (ie, news references to China). This suggests that Italian media coverage follows internal evolution more closely than global evolution, in contrast to other countries. This is probably due to the fact that Italy is the location of the first COVID-19 outbreak outside Asia. This observation is supported by Figure 2, which shows the citation share of Italian locations by Italian news media before and after the first COVID-19 death was confirmed in Italy on February 23, 2020. After this date, Italian locations represent about 74% of all places cited by Italian media (in our data set), with an increase of 45% with respect to the same statistics calculated before. Similar effects, although generally less intense, were observed in the other countries. Therefore, while media coverage is generally well synchronized with global COVID-19 incidence, the media attention gradually shifts toward the internal evolution of the pandemic as soon as domestic outbreaks erupt.

**Table 1:** Country-specific Pearson correlation coefficients for news coverage and global and domestic COVID-19 incidence, volumes of Reddit comments, and volumes of Wikipedia page views; domestic COVID-19 incidence and volumes of Reddit comments and Wikipedia views; and global COVID-19 incidence and volumes of Reddit comments and Wikipedia views.

Country		Global incidence of COVID-19	*P* value	Country incidence of COVID-19	*P* value	Reddit comments	*P* value	Wikipedia page views	*P* value
**Italy**									
	News	0.59	.04	0.92	<.001	0.43	.17	0.71	.009
	Global incidence of COVID-19	1	N/A^a^	—^b^	N/A	–0.42	.18	–0.01	.97
	Country incidence of COVID-19	—	N/A	1	N/A	0.30	.34	0.64	.02
**United Kingdom**									
	News	0.83	<.001	0.74	.006	0.50	.10	0.62	.03
	Global incidence	1	N/A	—		–0.04	.90	0.09	.77
	Country incidence	—	N/A	1	N/A	–0.15	.64	–0.04	.91
**United States**									
	News	0.84	<.001	0.79	.002	0.70	.01	0.64	.03
	Global incidence	1	N/A	—	N/A	0.25	.44	0.17	.60
	Country incidence	—	N/A	1	N/A	0.16	.62	0.08	.81
**Canada**									
	News	0.82	.001	0.71	.01	0.73	.007	0.59	.04
	Global incidence	1	N/A	—	N/A	0.23	.46	0.06	.85
	Country incidence	—	N/A	1	N/A	0.05	.87	–0.10	.76

^a^N/A: not applicable.

^b^—: not determined.

To more systematically explore the relationship between media coverage, public attention, and epidemic progression, we considered a linear regression model to nowcast the collective public attention for each country (quantified by the number of comments by geolocalized Reddit users or visits to relevant Wikipedia pages) using the volume of media coverage or the COVID-19 incidence as independent variables. We also included “memory effects” on the public attention by considering an exponential decaying term in the news time series [22]. We compared the three models, where the independent variables are the domestic incidence, the news volume, and the news volume plus a memory term, using the adjusted coefficient of determination (*R*^2^) [65]. We found that the model that considered only COVID-19 incidence performed worse than the models that considered media coverage (Table 2). This enforces the idea that collective attention is mainly driven by media coverage rather than by COVID-19 incidence. In addition, we found that including memory effects improved the model performance.

**Table 2:** Adjusted *R^2^* values for the three linear regression models applied to predict Reddit comments and Wikipedia page views (*P*<.001).

Country	Model I		Model II		Model III	
	Reddit comments	Wikipedia page views	Reddit comments	Wikipedia page views	Reddit comments	Wikipedia page views
Italy	0.52	0.65	0.68	0.73	*0.82* ^a^	*0.79*
United Kingdom	0.27	0.27	0.72	0.74	*0.82*	*0.85*
United States	0.42	0.35	0.82	0.74	*0.89*	*0.82*
Canada	0.35	0.23	0.83	0.71	*0.90*	*0.82*

^a^Italics indicate the superior performance of Model III.

More formally, we compared Model I to Model III using the Cox test [77] for nonnested models, and we compared Model II to Model III using the *F* test [78] for nested models. In all cases we obtained *P* values <.001, providing strong statistical evidence that Model III actually outperforms the other models. Not surprisingly, the coefficients of the “memory effects” term reported in Table 3 are negative for all countries. This implies that public attention actually saturates in response to news exposure and enables us to quantify the rate at which this phenomenon occurs.

In the next section, we will characterize the media coverage and internet users’ response more specifically in terms of content produced and consumed.

**Table 3:** Coefficient estimates (95% CI) for Model III (news plus memory effects). All coefficients are significant with *P*<.001.

Country	News		News plus memory effects	
	Reddit comments	Wikipedia page views	Reddit comments	Wikipedia page views
Italy	0.87 (0.60 to 1.14)	0.43 (0.29 to 0.58)	–0.41 (–0.59 to –0.23)	–0.15 (–0.26 to –0.04)
United Kingdom	0.95 (0.62 to 1.27)	0.99 (0.68 to 1.30)	–0.44 (–0.71 to –0.18)	–0.47 (–0.70 to –0.23)
United States	1.03 (0.79 to 1.27)	0.83 (0.58 to 1.09)	–0.51 (–0.77 to –0.24)	–0.46 (–0.73 to –0.19)
Canada	1.12 (0.89 to 1.36)	1.06 (0.67 to 1.44)	–0.40 (–0.59 to –0.22)	–0.45 (–0.72 to –0.18)

**Dynamics of Content Production and Consumption**

While collective attention and media coverage are well correlated in terms of volume, the content and topics discussed by media and consumed by internet users may not be as synchronized [79,80]. To shed light on this issue, we adopted an unsupervised topic modeling approach to extract prevalent topics in the news articles mentioned and discussed on Reddit. Indeed, users on Reddit often post submissions containing news articles, and discussion unfolds in the comments under the submissions. Differently from the previous section and to provide a comprehensive overview of the topics discussed, in this section, we do not take any geographical context into account. However, in Multimedia Appendix 1, we provide some additional insights into the specific topics discussed by users in different countries.

**Figure 3:**
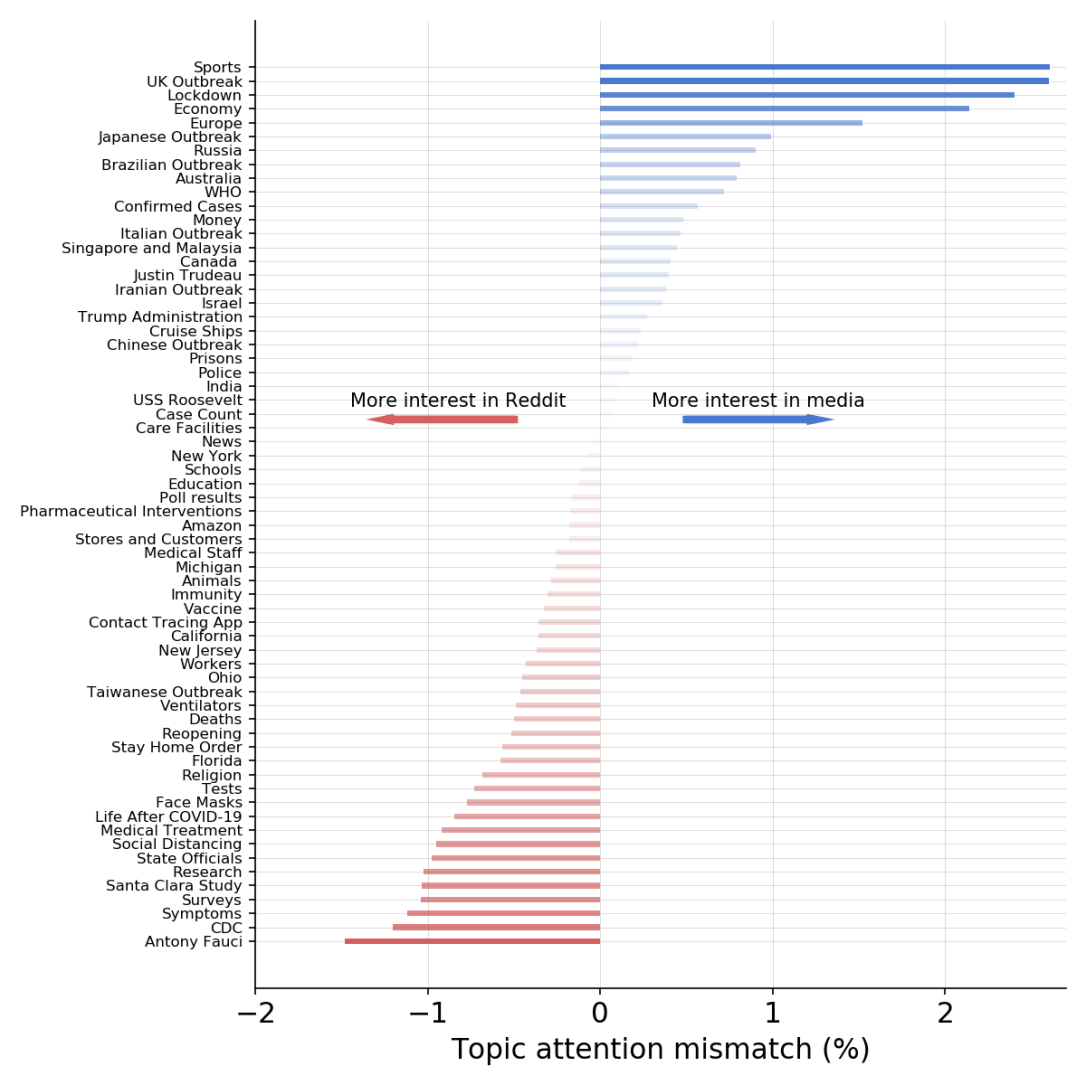
Differences in interest percentage shares of different topics by traditional media outlets and Reddit users. For example, +2% on the x-axis indicates that traditional media dedicates proportionally 2% more attention to that specific topic than Reddit users. CDC: US Centers for Disease Control and Prevention; UK: United Kingdom; WHO: World Health Organization.

We characterized the main topics discussed on Reddit by considering all submissions that included a news article in English. We then applied a topic modelling approach to the content of this news article set. Specifically, we extracted topics using NMF [67], a popular method for this type of task. In this way, we extracted the 64 most relevant topics in the news articles shared on Reddit. As a second step, we applied the model trained on the Reddit news to the set of articles published by mainstream media. That is, we characterized the news published by media outlets in terms of the topics discussed on Reddit. This choice enabled us to directly compare the topics covered by the media to the public discussion around this news exposure. A complete list of the 64 topics extracted with the most frequent words is provided in Multimedia Appendix 1. We considered the number of articles published on a certain topic as a proxy of general interest of traditional media in that topic; meanwhile, we measured the collective interest of Reddit users by the number of comments under the news articles on a specific topic. Figure 3 shows an overview of the topics extracted and a comparison of the interest of media and Reddit users. We obtained a diverse and heterogeneous set of topics, including the global spread of the virus (Outbreaks, WHO [World Health Organization], CDC [US Centers for Disease Control and Prevention]); COVID-19 symptoms, treatment, hospitals and care facilities (Symptoms, Medical Treatment, Medical Staff, Care Facilities); the economic impact of the pandemic and responses from the governments to the upcoming crisis (Economy, Money); different societal aspects (Sports, Religious Services, Education); and possible interventions to mitigate the spread of the virus (Face Masks, Social Distancing, Tests, Vaccine).

Overall, the levels of attention of traditional media outlets and Reddit users toward the different topics are in good accordance. Indeed, in Figure 3, we represent the difference between interest shares toward different topics in media and Reddit submissions. That is, we computed the percentage share of attention dedicated by news outlets and Reddit users to each topic, and we subtracted these two quantities. We observed a maximum absolute mismatch in interest share of 2.61%. However, we observed that Reddit users are slightly more interested in topics regarding health (Symptoms, Medical Treatment), nonpharmaceutical interventions and personal protective equipment (Social Distancing, Face Masks), studies and information on the epidemic (Research, Surveys, Santa Clara Study, CDC), and specific public figures such as Anthony Fauci. Interestingly, the Santa Clara Study topic refers to the discussion about a controversial scientific paper suggesting that a much higher fraction of the population in Santa Clara County was infected with respect to what was originally thought [81]. Because the study suggested a lower mortality rate, the preprint was quickly leveraged to support protest against lockdowns [82]; meanwhile, substantial flaws have been detected in the scientific methodology of the paper [83].

**Figure 4:**
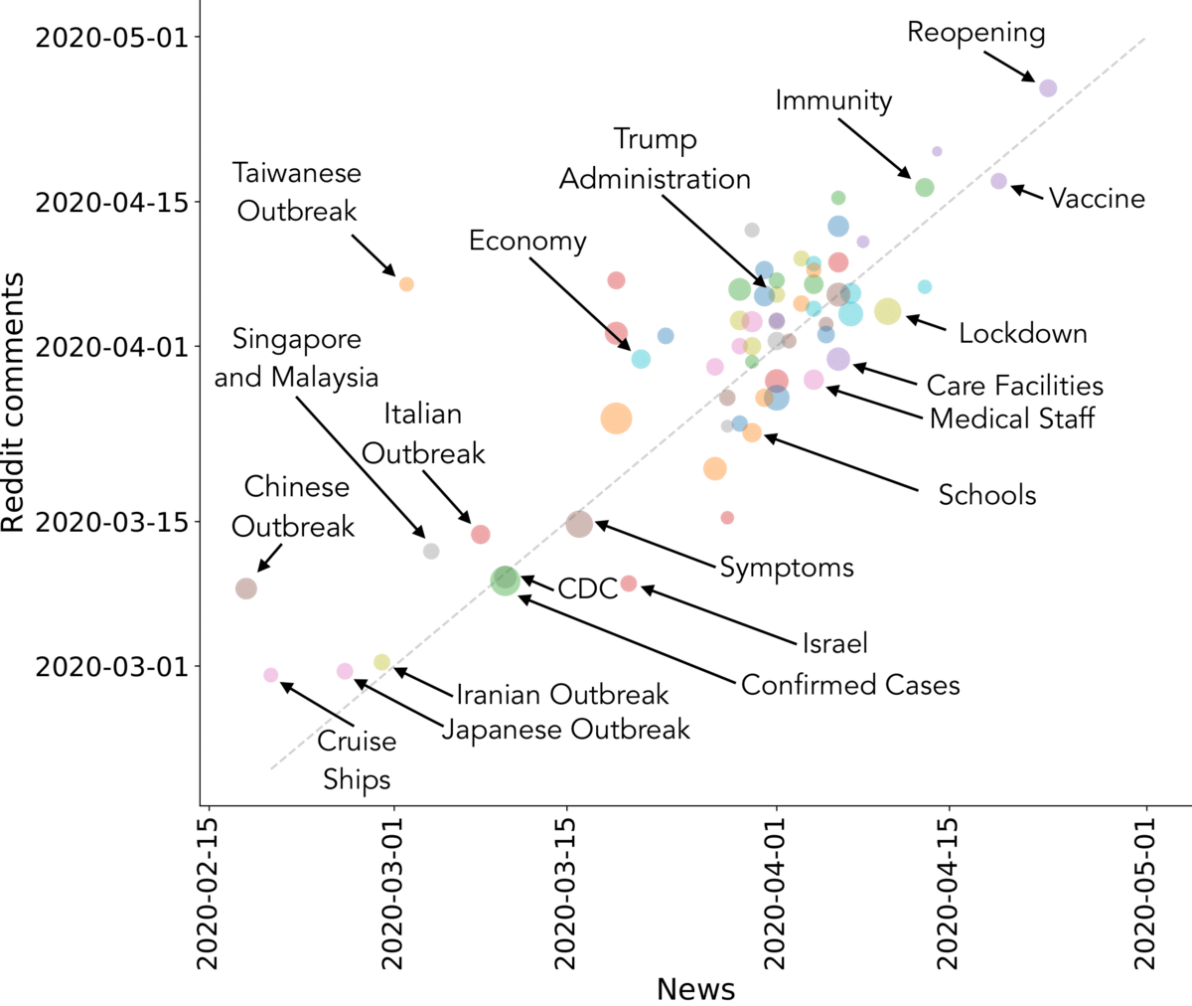
Scatter plot with the 64 topics extracted via nonnegative matrix factorization. The x-axis and y-axis coordinates indicate when a topic achieved 50% of its relevance in news outlets and on Reddit, respectively, during our analysis interval. CDC: US Centers for Disease Control and Prevention.

The overview of topics presented here does not take temporal dynamics of interest into account. However, topics showing similar overall statistics may present a mismatch in temporal patterns. Hence, in the following, we take into account the temporal evolution of interest toward different topics. In Figure 4, we represent each topic as a single point: the x-coordinate and y-coordinate indicate the *t*_1/2_ when the topic reached 50% of its total relevance R in news outlets and on Reddit, respectively, during the analysis interval. Therefore, topics in the bottom left region became relevant very early in the public discussion. Among these, we recognize themes centered on early COVID-19 outbreaks (ie, Chinese, Japanese, Iranian, and Italian outbreaks), events related to cruise ships, specific countries (ie, Israel, Singapore, and Malaysia), and topics regarding (early) health issues (ie, Symptoms, Confirmed Cases, and CDC). In contrast, topics in the top right region became relevant toward the end of the analysis interval (early May). Reasonably, this region contains topics about the resumption of activities after lockdown (ie, Reopening), the feasibility and timing of a possible vaccine against SARS-CoV-2 (ie, Vaccine), and discussions regarding acquired immunity and antibody tests (ie, Immunity). All other topics are clustered around the end of March and mid-April 2020, which is the period in which the general discussion surrounding the COVID-19 pandemic increased sharply, as also shown in Figure 1.

Note that the diagonal in Figure 4 (plotted as a dashed line) separates topics according to their temporal evolution. Above and below the diagonal, we find topics in which interest on Reddit grows slowly and quickly, respectively, with respect to the media coverage. Therefore, above the diagonal, the interest of Reddit users is mainly triggered by media exposure, while below it, the interest grows faster and declines rapidly despite sustained media exposure. The top left and bottom right regions are empty, indicating that as a first approximation, temporal patterns of attention by traditional media and Reddit users are well synchronized; however, interesting deviations from the diagonal are observable. For example, above the diagonal, one can mainly find topics related to various outbreaks, economics, and politics, for which the interest on Reddit follows the media coverage. Below the diagonal, we observe topics more related to everyday life, such as Schools, Medical Staff, Care Facilities, and Lockdown, for which the attention on Reddit accelerates with respect to media coverage and then declines rapidly. Note that our view of the topics discussed on Reddit is limited, as we only considered topics from news articles shared in submissions and did not explicitly take content expressed in comments into account. This ensures a proper comparison with the topics extracted from published news reports and explains the absence of points in the bottom right corner of Figure 4. 

## Discussion

### Principal Results

In this work, we characterized the response of internet users to both media coverage and COVID-19 pandemic progression. As a first step, we focused on the impact of media coverage on collective attention in different countries, characterized as volumes of country-specific Wikipedia page views and comments of geolocalized Reddit users. We showed that collective attention was mainly driven by media coverage rather than epidemic progression, rapidly became saturated, and decreased despite media coverage and COVID-19 incidence remaining high. These results are in very good accordance with findings obtained in previous contexts related to epidemics and pandemics. Indeed, a similar media-driven spiky unfolding of public attention, measured through the information-seeking and public discussions of internet users, was observed during the 2009 H1N1 influenza pandemic [84,85], the 2016 Zika virus outbreak [86], influenza season [87], and more localized public health emergencies such as the 2013 measles outbreak in the Netherlands [88]. Our findings confirm the central role of the media, showing how media exposure is capable of shaping and driving collective attention during a national and global health emergency. Media exposure is another important factor that can influence individual risk perception as well [79,89-91]. The timing and framing of the information disseminated by media can actually modulate the attention and, ultimately, the behavior of individuals [2]. This becomes an even greater concern in a context where the most effective strategy to fight the spread of disease involves containment measures based on individuals’ behavior.

Also, we showed how media coverage sharply shifted to the domestic situation as soon as the first death was confirmed in the home country. Arguably, this may have played an important role in individual risk perception. We can speculate that reframing the emergency within a national dimension can amplify the perceived susceptibility of individuals [92,93] and thus increase the adoption of behavioral changes [4,94]. Indeed, previous studies showed that at the beginning of February 2020, people were overly optimistic regarding the risks associated with the new virus circulating in Asia, and their perception sharply changed after the first cases were confirmed in their countries [9,95].

As a second step, we focused on the dynamics of content production and consumption. We modeled topics published in mainstream media and discussed on Reddit, showing that Reddit users were generally more interested in health, data regarding the new disease, and interventions needed to halt the spreading with respect to media exposure. By taking into account the dynamics of the extracted topics, we showed that while their temporal patterns are generally synchronized, the public attention to topics related to politics and economics is mainly triggered by media exposure, while the interest in topics more related to daily life increases on Reddit with respect to media coverage.

### Limitations

Of course, our research comes with limitations. First, we characterized the exposure of individuals to the COVID-19 pandemic by considering only news articles and YouTube videos published on the internet by major news outlets. However, individuals are also exposed to relevant information through other channels, with television being the most important [96]. Second, a 2013 Pew Internet Study found that Reddit users are more likely to be young men [97]; it was shown that around 15% of male internet users aged 18 to 29 years report using Reddit, compared to 5% of women in the same age range and 8% of men aged 30 to 49 years. Similarly, informal surveys proposed to users [98] showed that most respondents were males in their “late teens to mid-20s” and that female users were “very much in the minority.” Furthermore, Reddit is much more popular among urban and suburban residents than among individuals living in rural areas [97]. In addition to sociodemographic biases, other studies have suggested that Reddit has become an increasingly self-referential community, reinforcing the tendency to focus on its own contents rather than external sources [99]. Thus, the perceptions, interests, and behaviors of Reddit users may differ from those of the general population. A similar argument can be raised for Wikipedia searches. Indeed, the use of the internet, especially for information-seeking purposes, can vary across people with different sociodemographic backgrounds [100-102]. Additionally, we extracted Reddit users’ geographic location using a method based on regular expressions that has been successfully used in previous work [50]. However, because we have no ground truth data for comparison, we must consider the quality of location detection to be a possible limitation. Finally, our view on internet users’ reactions is partial. Indeed, we did not consider other popular digital data sources, such as Twitter. The reasons for this choice are twofold. First, many studies have already characterized public response during current and past health emergencies through the lens of Twitter [25,58,60,85,86,103,104]. Second, several studies have reported a high prevalence of bots as drivers of low-quality information and discussions on COVID-19 on this platform [24,25,105-107]. Thus, careful and challenging additional steps would be necessary to isolate, identify, and distinguish organic Twitter discussions and reactions that originated from traditional media from those sparked by social bots. We leave this for future work.

### Conclusions

Our work offers further insights to interpret public response to the current global health emergency and raises questions about possible undesired effects of communication. On one hand, our results confirm the pivotal role of media during health emergencies, showing how collective attention is mainly driven by media coverage. Therefore, because people are highly reactive to the news they are exposed to at the beginning of an outbreak, the quality and type of information provided may have critical effects on risk perception and behaviors, which will ultimately affect the unfolding of the outbreak. However, we also found that collective internet attention saturates and declines rapidly, even when media exposure and disease circulation remain high. Attention saturation has the potential to affect collective awareness and perceived risk, which ultimately affects the propensity toward virtuous individual behavioral changes aimed at mitigating the spread of disease. Furthermore, especially in the case of unknown viruses, attention saturation may exacerbate the spreading of low-quality information, which is likely to spread in the early phases of the outbreak when the characteristics of the disease are uncertain. Future work is needed to characterize the actual effects of attention saturation on human perceptions during a global health emergency. Our findings suggest that public health authorities should consider reinforcing specific communication channels, such as social media platforms, to compensate for the natural phenomenon of attention saturation. Indeed, these channels have the potential to create more durable engagement with people through a continuous loop of direct interactions. Currently, public health authorities are regularly issuing declarations on social media. However, the CDC did not even have a Twitter account in 2009 during the H1N1 pandemic (the account was created in May 2010). While this is just one example, it underlines that the communication of these global health emergencies through social media platforms is relatively new. Therefore, there is great need to further reinforce these channels and engage people through them. Simultaneously, public health authorities should consider strengthening additional communication channels. One example of this is the participatory surveillance platforms that are appearing worldwide, such as Influenzanet, Flu Near You, and FluTracking [108-110], which can deliver in-depth targeted information to individuals during public health emergencies and promote the exchange of information between people and public health authorities; this has potential to enhance the level of engagement in the community [111].

## Appendix

Multimedia Appendix 1 Supplementary information.

## Abbreviations

API: application programming interface
CDC: US Centers for Disease Control and Prevention
LDA: latent Dirichlet allocation
NMF: nonnegative matrix factorization
TF-IDF: term frequency–inverse document frequency
WHO: World Health Organization
