# Supplementary material for: Collective Response to Media Coverage of the COVID-19 Pandemic on Reddit and Wikipedia: Mixed-Methods Analysis
Source: J Med Internet Res. 2020 Oct 12;22(10):e21597. doi: 10.2196/21597 (PMC7553788; doi:10.2196/21597)
Supplement: Multimedia Appendix 1 [file jmir_v22i10e21597_app1.docx]

## Supplementary Information

## Nicolò Gozzi^1^, Michele Tizzani^2^, Michele Starnini^2^, Fabio Ciulla^3^, Daniela Paolotti^2^, André Panisson^2, ∗^ and Nicola Perra^1, †^

^1^ Networks and Urban Systems Centre, University of Greenwich, London, UK

^2^ ISI Foundation, Turin, Italy

^3^ Quid Inc., San Francisco, USA

^*^ [andre.panisson@isi.it](mailto:andre.panisson@isi.it)

**^†^** n.perra@greenwich.ac.uk

# Collective response to the media coverage of COVID-19 Pandemic on Reddit and Wikipedia

In this Supplementary Information we provide additional information on the linear regression models, the list of sources used and sensitivity analysis for the topic modeling. We also provide an insight on the topics discussed by Reddit users in different countries.

**Linear Regression – Diagnostic**

In the main text we use a Liner Model to nowcast the amount of Reddit discussion and Wikipedia page views using COVID-19 incidence and media coverage as independent variables. Here, we test model assumptions of homoscedasticity and no-autocorrelation of residuals. Homoscedasticity of residuals is assessed with the Breusch-Pagan Lagrange Multiplier test [1] on a fitted Ordinary Least Square model (OLS). Assuming a significance level $\alpha=0.05$, we obtain heteroscedasticity of residuals for Model II and Model III (Table 1).

Table 1. p-values for the Breusch-Pagan Lagrange Multiplier test on heteroscedasticity. The null hypothesis is that residuals are homoscedastic, hence a p-value < 0.05 indicates heteroscedasticity.

|  | *Model I* | | *Model II* | | *Model III* | |
| --- | --- | --- | --- | --- | --- | --- |
|  | reddit | wikipedia | reddit | wikipedia | reddit | wikipedia |
| Italy | 8.1E-02 | 2.4E-01 | 1.3E-08 | 2.3E-02 | 7.3E-08 | 3.2E-02 |
| UK | 2.9E-01 | 2.4E-01 | 2.7E-05 | 1.1E-07 | 4.6E-04 | 3.9E-06 |
| US | 1.5E-01 | 1.7E-01 | 7.2E-11 | 1.4E-07 | 3.2E-06 | 1.5E-04 |
| Canada | 3.4E-02 | 2.2E-01 | 7.5E-10 | 3.5E-07 | 6.7E-10 | 1.1E-07 |

We check for the autocorrelation of residuals in Figure 1, where we plot the partial auto-correlation function for Model III (analogous results are obtained for Model I and II). We notice a significant correlation at lag 1 in all cases, and in some cases also at higher orders (up to lag 16).

**
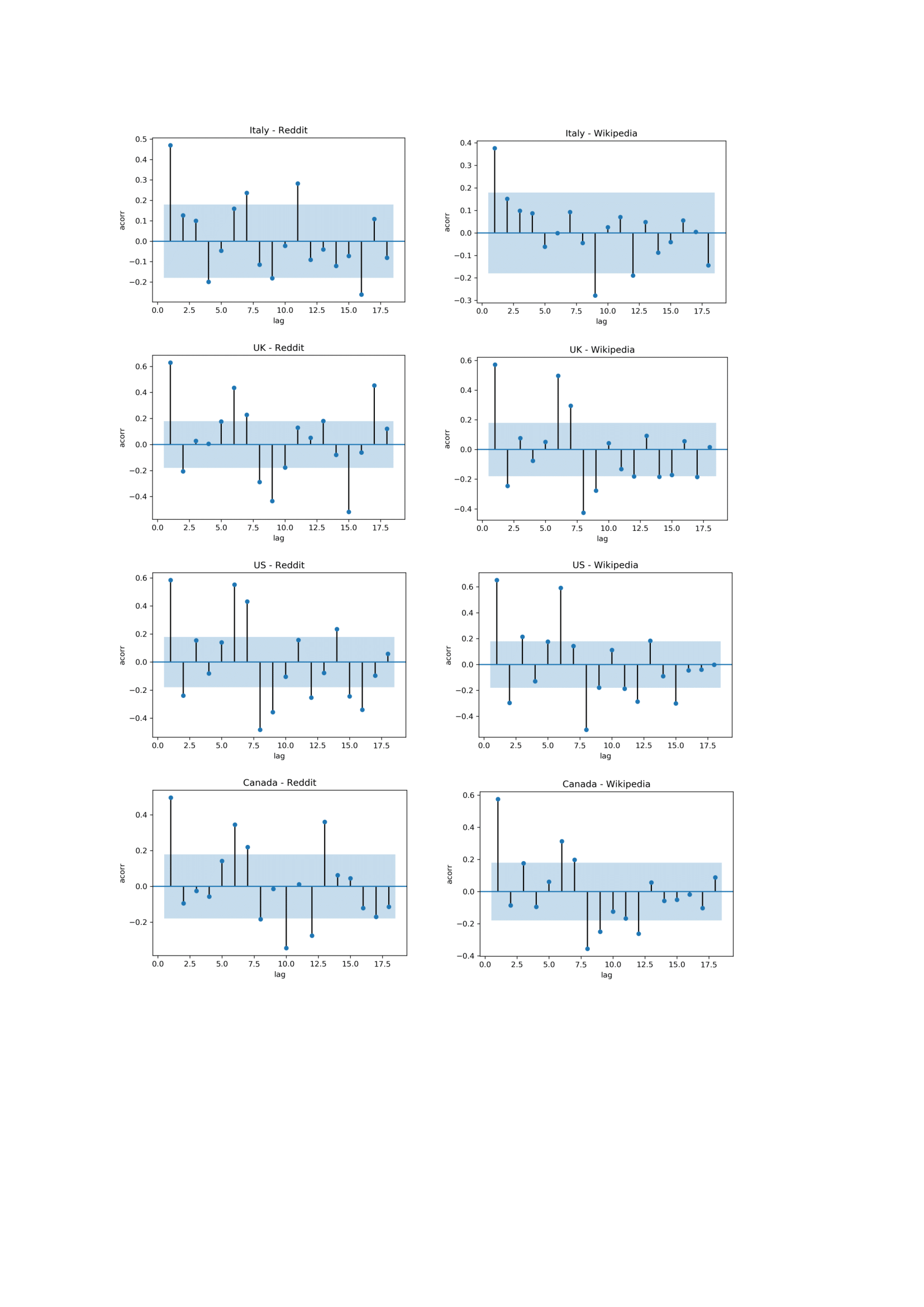
**

Figure 1. Autocorrelation plot for Model III for different lags values with 90% confidence intervals.

Heteroscedasticity and autocorrelation of residuals are common issues in time series regression. In this case, while OLS is still unbiased, variances are not valid anymore. We fix this issue operating a correction using the Newey–West estimator [2]. This method allows to estimate a covariance matrix which is robust to both heteroscedasticity and autocorrelation of residuals up to lag p (which is set looking at the correlograms in Fig. 1).

**Topic Modeling – Number of Topics**

Finding a reasonable number of topics K and quantifying the coherence of the extracted topics is a long-standing problem, that can be approached either using human evaluation or using other quantitative measures [3, 4]. In the non-negative matrix factorization (NMF) framework, model goodness of fit is measured through the reconstruction error $\left\| \mathbf{X}-\mathbf{WH} \right\|_{F}^{2}$. A large number of topics K results in lower reconstruction error, with the risk of overfitting and loss of topic coherence. At the same time, a small number of topics might underfit the data and only partially capture the topics present in the documents, since NMF does not enforce a document to be associated to a topic (i.e., $\sum_{k} w_{ik}$might be zero for some document i). We can measure the capacity of the model to capture the topics present in the documents through the topic strength, i.e., $\sum_{i, k} w_{ik}$. We recall that topic strength is also used in the main text for building the time series that represent the strength of topic k at time t, i.e., $\sum_{i\in D^{(t)}} w_{ik}$.

To avoid both overfitting and underfitting, we select a number of topics K that results in both high topic strength and high topic coherence. Among the many methodologies for quantifying topic coherence, the one described in [4] has been shown to give scores that are in good agreement with human evaluators. In our work, we use this methodology to compare the distribution of topic coherence for different choices of K, and then we use the word intrusion task described in [3] to manually assess the quality of the topics extracted by our choice of K.

The boxplots in Figure 2 show the distribution of topic coherence for a given K. For a given topic model with K topics, we extract the top 10 words for each topic. Then, for each topic, coherence is calculated using a combination of indirect cosine measure, normalized pointwise mutual information, and a boolean sliding window of 110, using the implementation provided by Gensim [5]. The average topic coherence for K=64 is 0.53, which is higher than lower values of K. Higher values of K result in a topic coherence distribution that is comparable to K=64, but include many topics with low coherence, which is a sign of possible overfitting. These results show that K=64 is a good balance between enough captured topic strength and good topic coherence.

In Figure 3 we show the results for the manual quality assessment of the topics extracted with K=64. The word intrusion task described in [3] was performed for the topics extracted with K=64. For each topic k, we select the 6 most representative words with highest $h_{kj}$, we add an intruder word $j'$ with small value $h_{kj'}$ for topic k but with high value for some other topic k', and we shuffle the words positions. We repeat this step 5 times for each topic and ask 4 different human annotators to find out which is the intruder word.

For all topics, precision is safely higher than the random baseline. The results show that for 48 out of 64 topics, the annotators were able to select the right intruder word in more than 80% of the times. Only for a few (3) topics the annotators were able to find the intruder word in less than 50% of the cases. Furthermore, for these topics the annotators’ errors were made most of the times on the same word. This implies that also in apparently less accurate topics, 5 of the 6 most representative words show a strong coherence.

Figure 2. Total topic strength $\sum_{i, k} w_{ik}$ captured by topic models with varying number of topics *K*, along with a boxplot with the distribution of topic coherence for all topics extracted for a given *K*. Each boxplot shows the median topic coherence (orange line), the interval containing 50% of the points (the box), the interval containing all points except outliers (the whiskers) and the outliers (small circles).


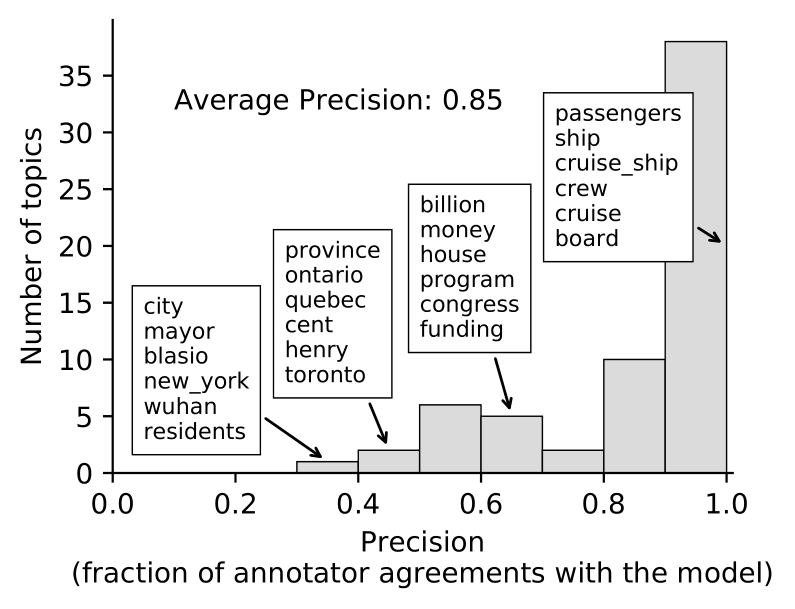


Figure 3. Results of the word intrusion test.

Table 2: List of 64 topics extracted using NMF with most frequent words.


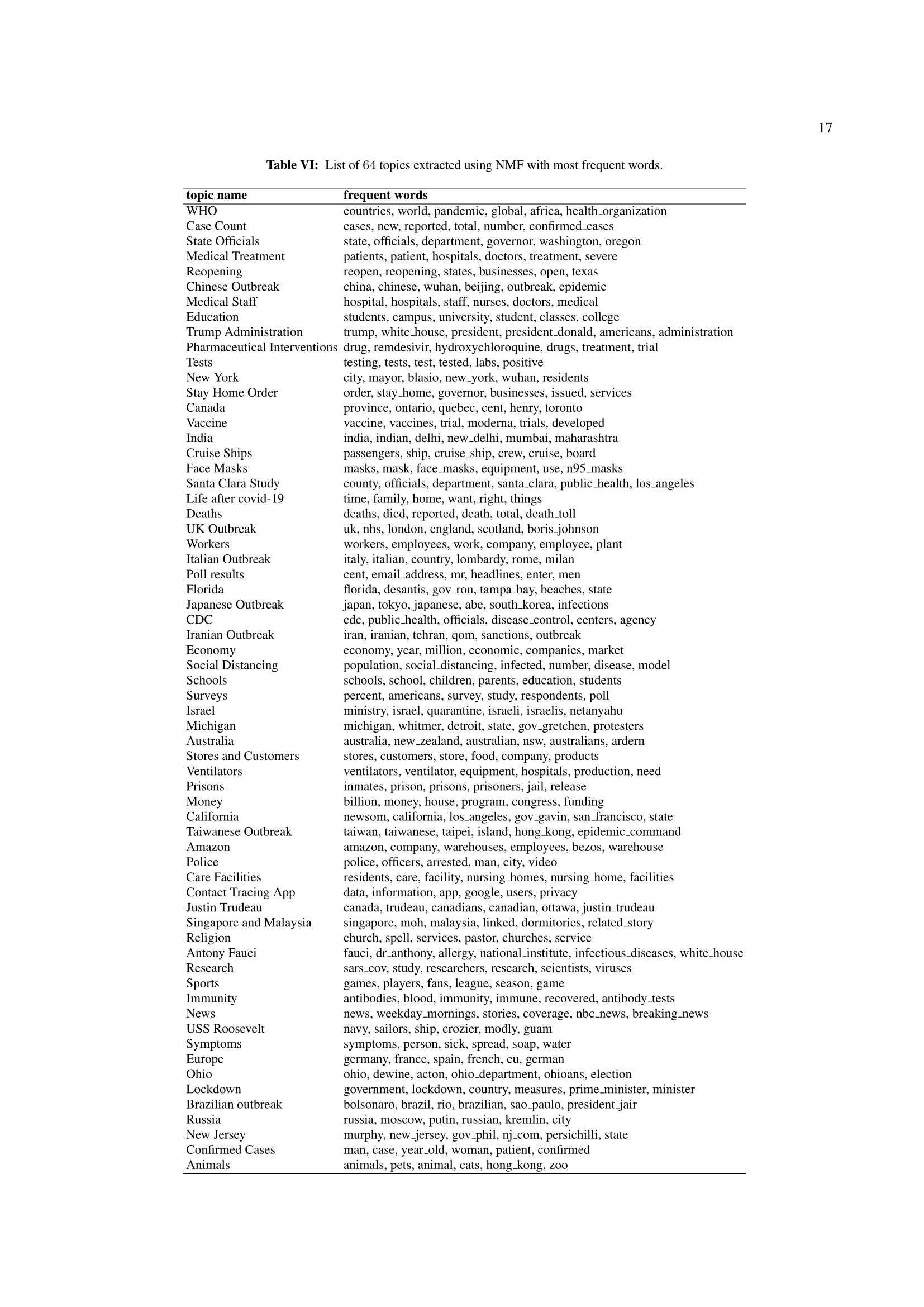


**Data Sources**

Table 3. Lists of news sources considered for Italy, United Kingdom, United States, and Canada. These lists aim to provide the most complete overview of the communication medias landscape in different countries, while facing with the limitation imposed by the API.


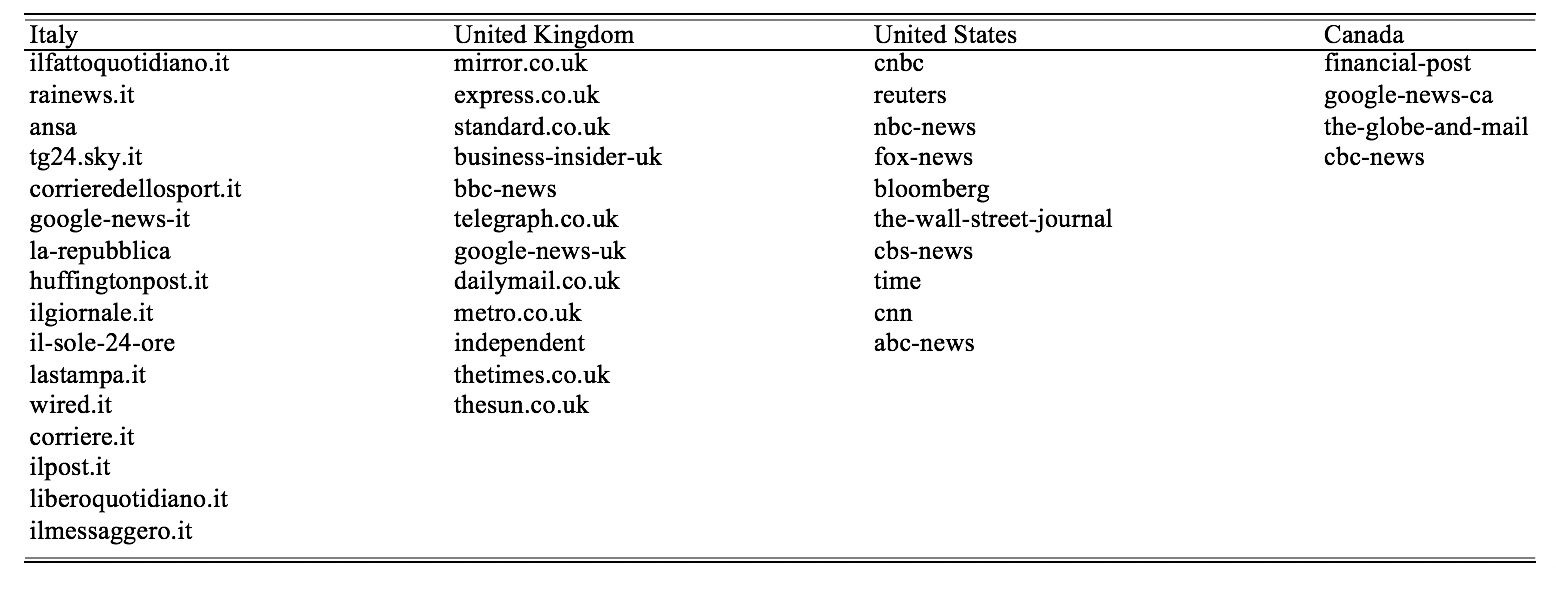


Table 4. Lists of YouTube channels considered for Italy, United Kingdom, United States, and Canada. In parentheses, we report the number of subscribers as of 2020/05/18.


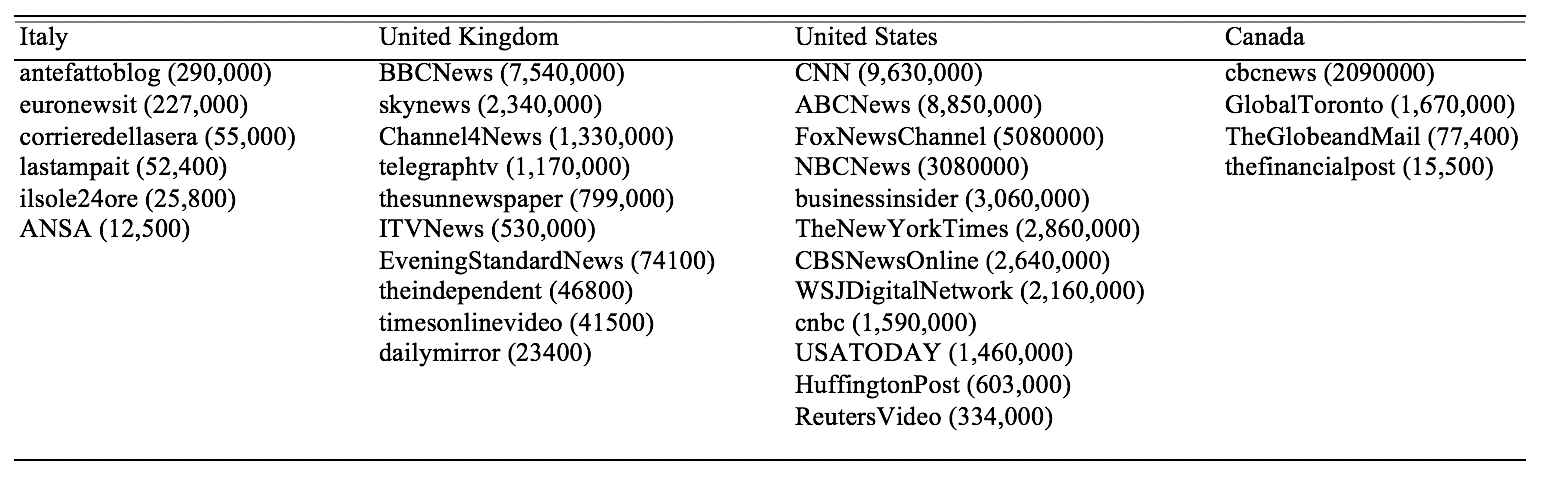


Table 5. List of Wikipedia articles related to the COVID-19


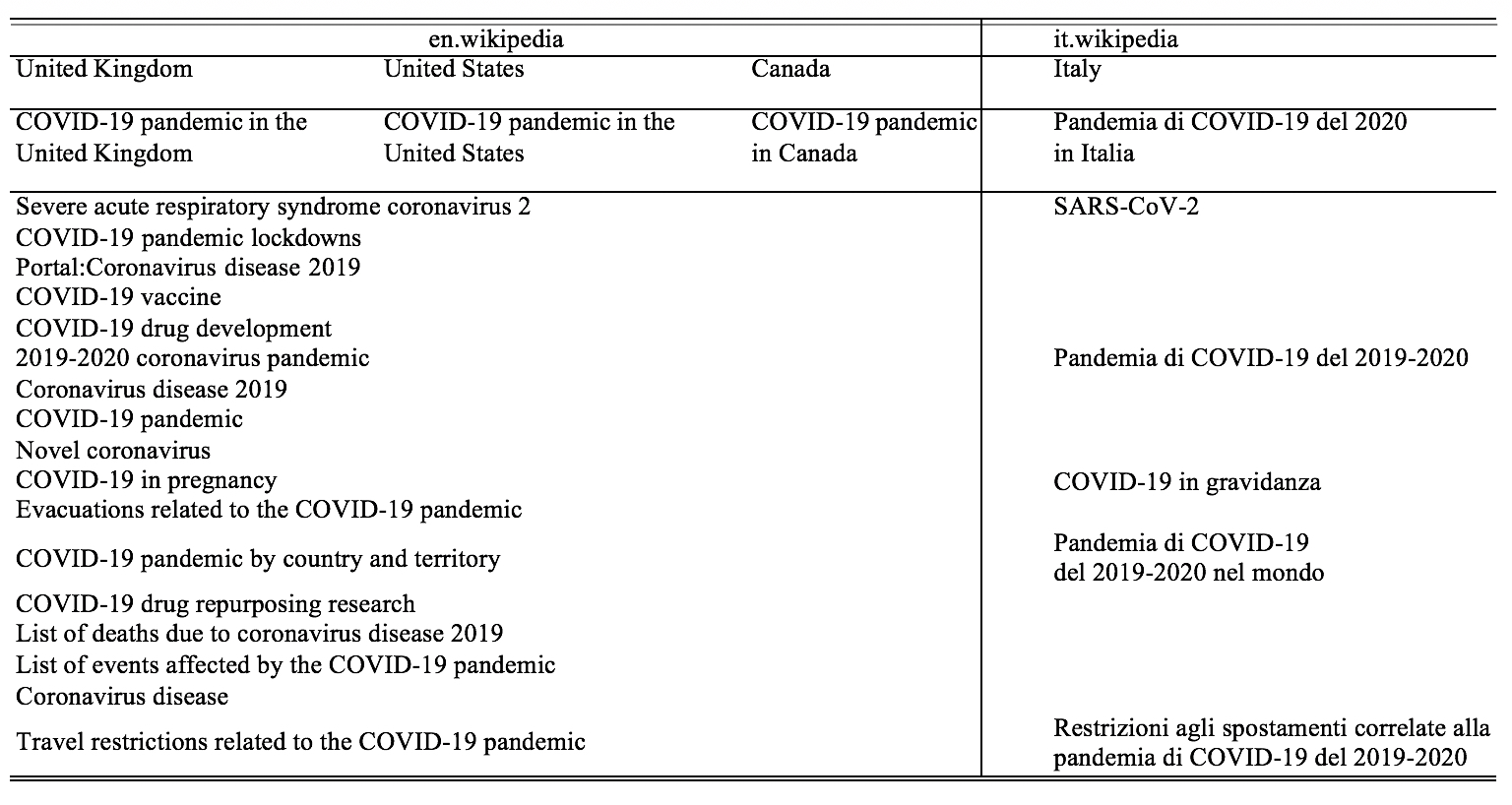


**Reddit Discussion in Different Countries**


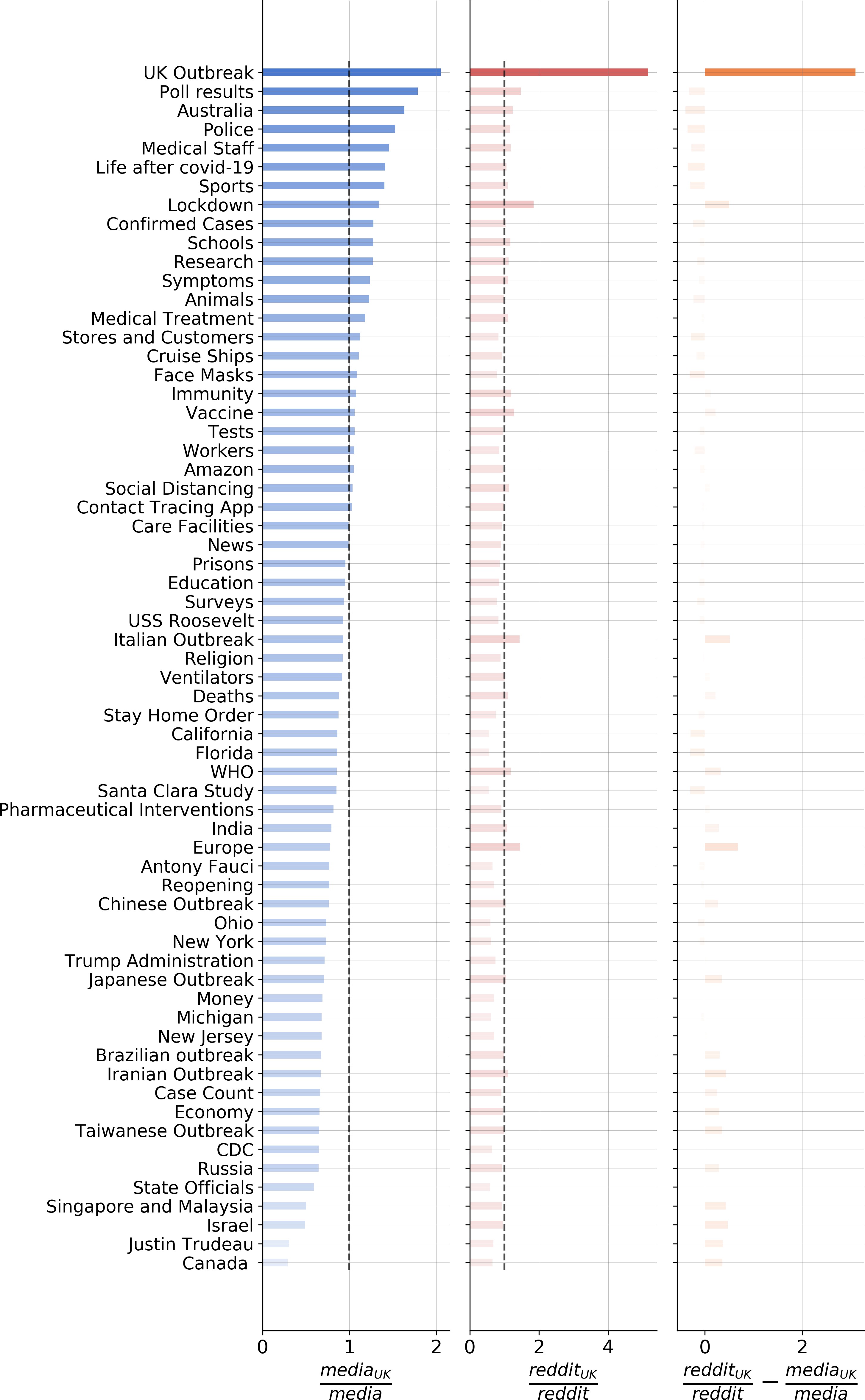


Figure 4: From left to right: ratio between UK media interest and general media interest for different topics; ratio between UK Reddit users’ interest and general Reddit users’ interest for different topics; differences between these two quantities for different topics. In the first two plots, topics to the left of the dashed line (on 1) are less discussed in by UK media/users with respect to the general discussion, while topics to the right are more discussed. In the last plot, positive (negative) bars indicated that UK Reddit users pay generally more (less) attention to that topic with respect to UK media.


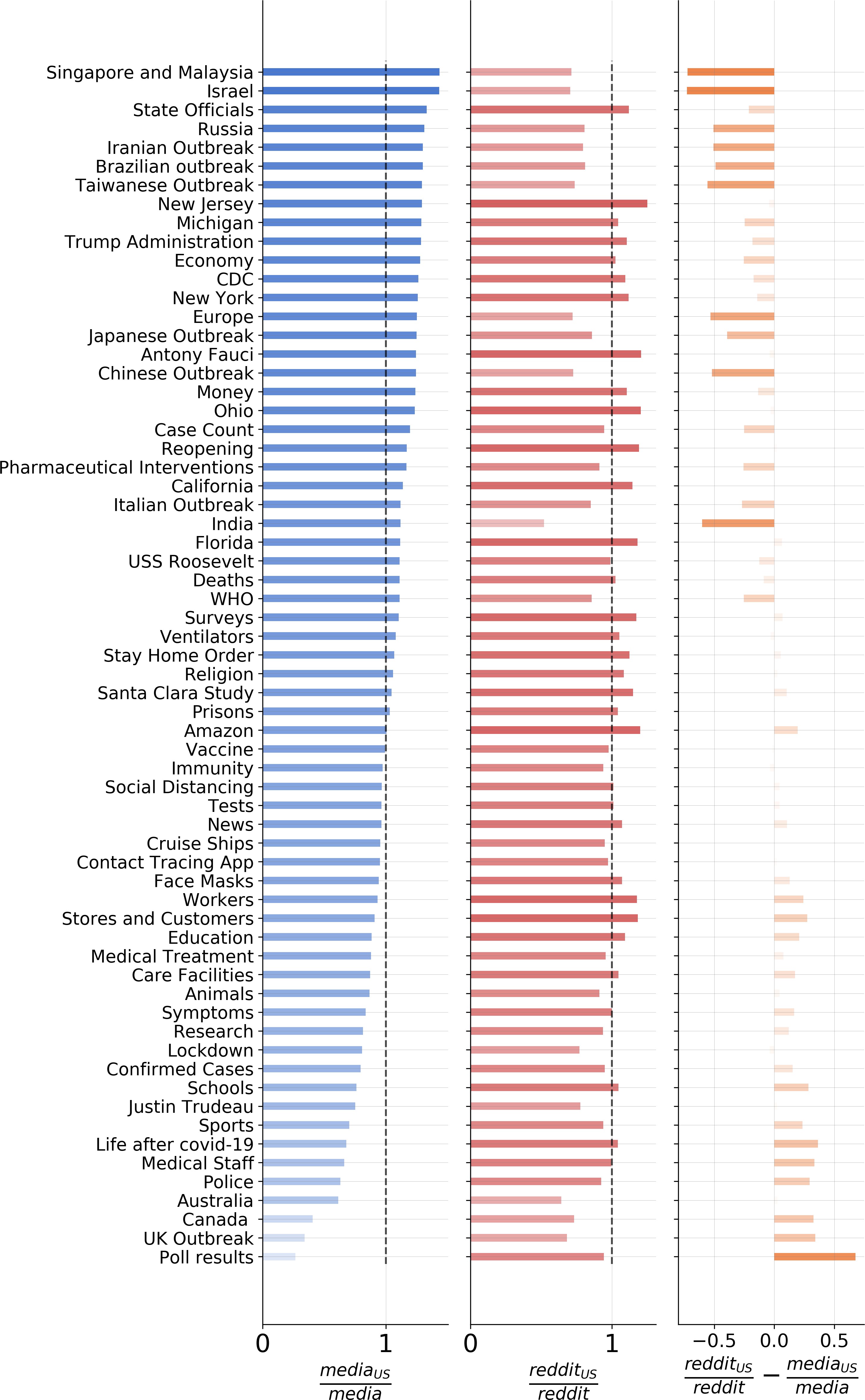


Figure 5: From left to right: ratio between US media interest and general media interest for different topics; ratio between US Reddit users’ interest and general Reddit users’ interest for different topics; differences between these two quantities for different topics. In the first two plots, topics to the left of the dashed line (on 1) are less discussed in by US media/users with respect to the general discussion, while topics to the right are more discussed. In the last plot, positive (negative) bars indicated that US Reddit users pay generally more (less) attention to that topic with respect to US media.


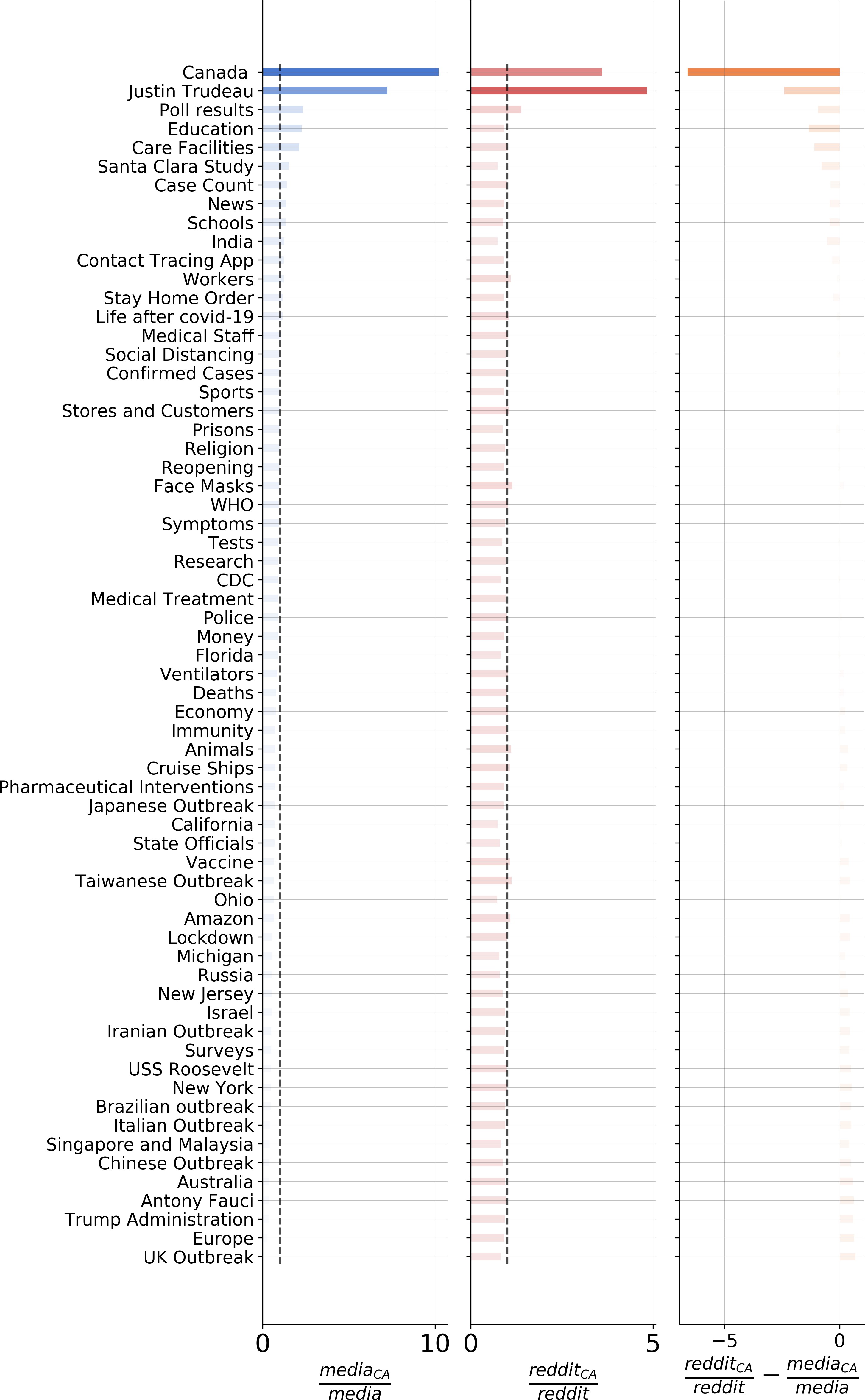


Figure 6: From left to right: ratio between Canadian media interest and general media interest for different topics; ratio between Canadian Reddit users’ interest and general Reddit users’ interest for different topics; differences between these two quantities for different topics. In the first two plots, topics to the left of the dashed line (on 1) are less discussed in by Canadian media/users with respect to the general discussion, while topics to the right are more discussed. In the last plot, positive (negative) bars indicated that Canadian Reddit users pay generally more (less) attention to that topic with respect to Canadian media.

## References

1. Breusch, T. S.; [Pagan, A. R.](https://en.wikipedia.org/wiki/Adrian_Pagan) (1979). "A Simple Test for Heteroskedasticity and Random Coefficient Variation". Econometrica. 47 (5): 1287–1294.
2. Newey, W. K., & West, K. D. (1987). A Simple, Positive Semi-definite, Heteroskedasticity and Autocorrelation Consistent Covariance Matrix. Econometrica, 55(3), 703–08.
3. Jonathan Chang, Sean Gerrish, Chong Wang, Jordan L Boyd-Graber, and David M Blei, “Reading tea leaves: How humans interpret topic models,” in Advances in neural information processing systems (2009) pp. 288–296.
4. Michael Röder, Andreas Both, and Alexander Hinneburg, “Exploring the space of topic coherence measures,” in Proceedings of the eighth ACM international conference on Web search and data mining (2015) pp. 399–408.
5. <https://radimrehurek.com/gensim/models/coherencemodel.html>
